# 17β-Estradiol Induces Mitophagy Upregulation to Protect Chondrocytes *via* the SIRT1-Mediated AMPK/mTOR Signaling Pathway

**DOI:** 10.3389/fendo.2020.615250

**Published:** 2021-02-03

**Authors:** Runhong Mei, Peng Lou, Guanchao You, Tianlong Jiang, Xuefeng Yu, Lei Guo

**Affiliations:** ^1^ Department of Orthopaedics, The Fourth Affiliated Hospital of Nanchang University, Nanchang, China; ^2^ Department of Orthopedics, The First Hospital of China Medical University, Shenyang, China

**Keywords:** 17β-estradiol, mitophagy, Sirtuin-1, AMP-activated protein kinase/mammalian target of the rapamycin signaling pathway, osteoarthritis

## Abstract

Increasing evidence reveals that estrogen, especially 17β-estradiol (17β-E2), is associated with articular cartilage metabolism disorder and postmenopausal osteoarthritis (OA). SIRT1, AMPK, and mTOR are regarded as critical mitophagy regulators. Recent studies have shown that mitophagy displays a protective effect against OA, but the molecular mechanism is not well known. This study aimed to investigate the effect of 17β-E2 on Sirtuin-1 (SIRT1) expression and the induction of mitophagy upregulation by 17β-E2 *via* the SIRT1-mediated AMP-activated protein kinase (AMPK)/mammalian target of the rapamycin (mTOR) signaling pathway to protect chondrocytes. ATDC5 chondrocytes were treated with different concentrations of 17β-E2 (0 M, 1 × 10^-9^ M, 1 × 10^-8^ M, and 1 × 10^-7^ M) for 24 h or pretreatment with or without NAM (SIRT1 inhibitor), Compound C (AMPK inhibitor) and S1842 (mTOR inhibitor) for 30 min prior to treatment with 17β-E2 (1 × 10^-7^ M) for 24 in each groups. Expression of SIRT1 was evaluated by real-time PCR, Western blotting and confocal immunofluorescence staining. Then, the mitophagosomes in cells were observed under a transmission electron microscopy (TEM), and the AMPK/mTOR signaling pathway was detected by Western blotting. The mitophagy-related proteins, p-AMPK, p-mTOR, p-JNK, and p-p38 were also identified by Western blot analysis. The chondrocytes viability and proliferation were determined by MTT and 5-Bromo-2’-deoxyuridine (BrdU) assay. These experiments were independently repeated 3 times The study found that 17β-E2 increased the expression level of SIRT1, p-AMPK, and mitophagy-related proteins but decreased p-mTOR expression, and then induced mitophagy upregulation in chondrocytes. More mitochondrial autophagosomes were observed in 17β-E2-treated chondrocytes under a transmission electron microscope. Also, 17β-E2 improved cell viability and proliferation with the higher expression of SIRT1 and activation of the AMPK/mTOR signaling pathway. However, SIRT1 inhibitor nicotinamide (NAM) and AMPK inhibitor Compound C blocked the beneficial effect of 17β-E2. In summary, this study was novel in demonstrating that 17β-E2 induced mitophagy upregulation to protect chondrocytes *via* the SIRT1-mediated AMPK/mTOR signaling pathway.

## Introduction

With the prevalence and incidence of osteoarthritis (OA) are expected to increase every year with the increase in life expectancy (1). OA has gradually become one of the most common chronic diseases in the world, affecting an estimated 10% of men and 18% of women over 60 years of age (2). Epidemiological studies have shown that there are significant sex differences in both the prevalence and incidence of OA (3, 4). OA is more prevalent in men than women before age 50. After menopause, the incidence of OA in women increases (5). Postmenopausal women have OA that affects multiple joints with greater severity, suggesting a chondroprotective effect of estrogen (6, 7). The characteristic pathological changes of OA include hyperplasia of the synovial membrane, degeneration of cartilage or exposure of the subchondral bone, wear and tear of the meniscus, and formation of osteophytes. Among the various pathological and physiological mechanisms of OA, the mechanisms associated with sex hormone regulation have gained much attention, especially those relevant to estrogen. Previous studies have shown that estrogen, especially 17β-E2, has a potential protective effect on chondrocytes (8).

Autophagy is a process of cell self-digestion that responds to different stress conditions, including the uptake of intracellular contents and degradation of the lysosome system (9, 10). Autophagy is necessary to maintain cell dynamic balance and viability by degrading aggregated proteins and impaired organelles, especially damaged mitochondria (11, 12). Mitochondria autophagy (mitophagy) is a form of selective autophagy involved in the removal of dysfunctional mitochondria through degradation in the autophagy–lysosomal system (13). The mitophagy function of chondrocytes in the articular cartilage of patients with OA is weakened, thus accelerating cell apoptosis and cartilage degeneration (14). However, the underlying mechanism of mitophagy regulation in OA is not fully understood.

Many studies have shown that sirtuin-1 (SIRT1) is an NAD^+^-dependent type III histone deacetylase involved in the occurrence and development of OA. Previous studies demonstrated that the expression of SIRT1 decreased with the development of OA, and the drop in SIRT1 expression in chondrocytes might lead to chondrocyte hypertrophy and cartilage matrix loss (15). In addition, Matsuzaki et al. found that the loss of SIRT1 in chondrocytes led to the accelerated development of mouse OA under mechanical pressure and during aging, indicating that SIRT1 prevented the development of OA (16). Therefore, SIRT1 is important in protecting chondrocytes and preventing the progression of OA. In addition, recent studies have shown the involvement of SIRT1 in the regulation of acetylation–deacetylation in autophagy (17). Some autophagy-related proteins microtubule-associated protein 1A/1Blight chain 3 (LC3), ATG5, and ATG7 are deacetylated by SIRT1 (18). A recent study showed that SIRT1-mediated autophagy had a protective effect on the apoptosis of chondrocytes (19).

AMP-activated protein kinase (AMPK) is considered to be a major metabolic energy receptor, which is activated when the cell energy charge decreases (AMP/ATP ratio increases), further regulating the dynamic balance of energy metabolism (20–22). Given its critical role in controlling energy homeostasis, AMPK has attracted widespread interest as a therapeutic target for potential metabolic diseases, including diabetes, obesity, tumors, and especially osteoarthritis. The decrease in mitochondrial biosynthesis in chondrocytes is related to the decrease in AMPK activity and the expression of SIRT1, PGC1α, and nuclear respiratory factor 1 (NRF1) (23). AMPK is a key molecule related to chondrocyte metabolism, which regulates energy metabolism through mediators such as SIRT1 and mammalian target of rapamycin (mTOR) (24). mTOR is one of the main inhibitors of autophagy. It is an important signaling molecule downstream of AMPK. An increase in phosphorylation can inhibit the levels of mTOR (25), thus enhancing autophagy. Moreover, SIRT1-mediated activation of AMPK leads to the inhibition of mTOR, which also propels autophagy (26, 27).

Although estrogen (17β-estradiol) has a potential protective effect on OA, the mechanism of 17β-E2 in OA is still unclear. Luca et al. indicated that many studies highlighted the importance of SIRT1 in pathophysiological processes and showed how it interacted with the AMPK and mTOR pathway (28). Shen et al. (29) reported that 17β-estradiol was able to protect cardiomyocytes from AngII-induced injury with a profound upregulation of SIRT1 and activation of AMPK. In a mouse OA model, estrogen may inhibit mTOR signaling pathway *via* activating ERK, which will promote chondrocytes autophagy to protect AMPK mutant mice from OA (30). In addition, estrogen at a pharmacological concentration could stimulate the production of type II collagen in the growth plate chondrocytes *in vitro*, thus promoting chondrogenesis (31). Although great efforts have been made, 17β-E2’s contribution to chondrocytes in osteoarthritis is still unclear.

This study provided a novel insight into how SIRT1 was involved in 17β-E2-mediate beneficial effect on chondrocytes in OA. It also suggested an improved understanding of the effect of 17β-E2 on the expression of SIRT1, mitophagy, and AMPK/mTOR pathway in ATDC5 chondrocytes. The findings of this study might provide a new potential target for the treatment of OA.

## Materials and Methods

### Chemicals

17β-Estradiol, 3-(4,5-dimethylthiazol-2-yl)-2,5-diphenyl-2H-tetrazol-3-ium bromide (MTT), 5-Bromo-2’-deoxyuridine (BrdU), and dimethyl sulfoxide (DMSO) were obtained from Abcam (MA, USA) and Promega (WI, USA).

### Reagents

The SIRT1 inhibitor (nicotinamide, NAM), AMPK inhibitor (Compound C), and 4,6-diamidino-2-phenylindole (DAPI) were obtained from Beyotime Biotechnology (Shanghai, China). The JNK inhibitor (SP600125), mTOR inhibitor (S1842), and p38 inhibitor (SB203580) were bought from Sigma–Aldrich (MO, USA). The polyclonal goat anti-mouse heat shock protein 60 (Hsp60) antibody (sc-1052) (1:200), the monoclonal mouse anti-mouse microtubule-associated protein 1A/1B-light chain 3 (LC3) antibody (sc-376404) (1: 100), rabbit monoclonal anti-β-actin and the polyclonal rabbit anti-mouse primary translocase of outer membrane (TOM) 20 antibody (sc-11415) (1: 200) were purchased from Sigma (MO, USA). The total/phosphor-p38 antibody and total/phosphor-JNK antibody were brought from Promega (WI, USA). TaqMan reagents and TRIzol reagent were obtained from Beyotime (Shanghai, China). The immunofluorescence staining kit was bought from Sigma.

### Cell Culture and Treatments

ATDC5 chondrocytes were obtained from Kebai (Nanjing, China). They were plated in six-well plates (Beyotime, Shanghai, China) at a density of 6 × 10^4^ cells/well and cultured at 37°C in 5% CO_2_/95% humidity. Under the circumstances of starvation, the chondrocytes were incubated with DMEM/F12 for 24 h after the confluence was 75% and then treated with 17β-E2. They were treated with incremental doses of 17β-E2 (0 M, 1 × 10^-9^ M, 1 × 10^-8^ M, and 1 × 10^-7^ M) for 24 h. For the combination treatments, the cells were pretreated with the corresponding inhibitors for 30 min prior to treatment with an optimum concentration of 17β-E2 (1 × 10^-7^ M) for 24 h. SIRT1 inhibitor (NAM) was used at 15 µM, AMPK inhibitor (Compound C) at 20 µM, mTOR inhibitor (S1842) at 10 µM, JNK inhibitor (SP600125) at 10 µM and p38 inhibitor (SB203580) at 20 µM (32–34). Finally, the chondrocytes were harvested for RT-PCR and Western blot analysis.

### Real-Time PCR Analysis

The RT-PCR was used to detect the level of SIRT1 mRNA in ATDC5 chondrocytes. TRIzol reagent (Invitrogen, Shanghai, China) was used to extract total RNA from chondrocytes, according to manufacturer’s instructions The RevertAid First-Strand cDNA Synthesis Kit was used to reverse-transcribe RNA into cDNA. Then, the mRNA levels were detected using appropriate specific primers and an RT-PCR Kit (Takara Bio, Beijing, China), with cDNA as a template. Forward and reverse primer sequences of all genes were as follows: SIRT1 forward (5′-AGTTCCAGCCGTCTCTGTGT-3′) and reverse (5′-GATCCTTTGGATTCCTGCAA-3′); and GAPDH forward (5′-ACATGACTACACTCTCGGTAAT-3′) and reverse (5′-TGTCTCGCTCCTAGAAGAAGTA-3′). The data were normalized to GAPDH, which was used as an internal reference control. The conditions for the PCR reactions were 95°C for 10 s, 95°C for 30 s, 60°C for 40 s and 72°C for 45 s, for a total of 40 cycles. The final data were analyzed by the 2−ΔΔCt method. Each experiment was conducted independently and repeated 3 times. The results are shown as mean ± SD.

### Immunostaining and Microscopy

The ATDC5 chondrocytes were seeded on 24-well plates and then pretreated with or without NAM for 30 min before incubated with 1 × 10^-7^ M 17β-E2 for 24 h. After incubation, the cells were washed with PBS 3 times, fixed with 4% PFA for 25 min, and permeated with 0.3% Triton X-100 for 15 min at room temperature. Then, the chondrocytes were blocked with 5% bovine serum albumin for 40 min and incubated with primary rabbit anti-SIRT1 antibodies (1:1000) or primary antibody anti-LC3 (1:200) at 4°C overnight. Subsequently, they were incubated with the secondary antibody employing a goat anti-rabbit IgG (1:3000) or a secondary antibody anti-DyLight 594 (1:200) for 60 min at room temperature. At last, they were incubated with DAPI for 5–10 min at room temperature. Ten visual fields were randomly selected for observation under a confocal laser scanning microscope, and then the fluorescence intensity was measured using ImageJ Software 1.48 (ML, USA).

### Protein Extraction and Western Blotting Analysis

Western blotting was performed as previously described (35). After each treatment, the chondrocytes were rinsed with PBS twice. Then, the chondrocytes collected by centrifugation were lysed in radio-immunoprecipitation assay buffer at 4°C for 25 min, and the lysates were centrifuged at 12,000 rpm for 15 min at 4°C. A Pierce BCA Protein Assay Kit was used to determine the protein concentration following the manufacturer’s protocols (Thermo Fisher Scientific, USA). SDS-PAGE (10% SDS) was performed on the same amount of protein samples. After electrophoresis, the protein was transferred to a nitrocellulose membrane and sealed with 5% skimmed milk powder containing TBST (0.05% Tween-TBS, Beyotime, China) at room temperature for 1 h. Then, the protein was rinsed with TBST 3 times. The membranes were incubated with the primary antibodies: anti-SIRT1, anti-AMPK, anti-p-AMPK, anti-LC3 anti-TOM20, anti-Hsp60, anti-JNK, anti-p-JNK, anti-p38, anti-p-p38, anti-mTOR, anti-p-mTOR or anti-β-actin antibodies. All results were normalized to β-actin protein concentration. After overnight incubation at 4°C, the membranes were rinsed and incubated with the corresponding secondary antibodies for 1 h at room temperature. After rinsing with TBST 3 times again, a BeyoECL plus kit (Beyotime, Shanghai, China) was applied to visualize the proteins.

### Transmission Electron Microscopy Observation

The chondrocytes were digested with trypsin and centrifuged at 1200*g* for 6 min. They were fixed in 2.5% phosphate-buffered glutaraldehyde and then post-fixed in 1% osmium tetroxide in water for 1 h. They were dehydrated with gradient acetone, embedded, and sectioned. The samples were double stained with uranyl acetate and lead citrate for 10 min. Then, the ultrastructures of cells were viewed under a JEM-1230 TEM (JEOL Ltd., Tokyo, Japan).

### Cell Viability Testing

MTT assay was used to evaluate the viability of chondrocytes subjected to different treatments. The chondrocytes were seeded in 96-well plates with a density of 1 × 10^4^ cells/well, which contained serum-free DMEM. After the attachment of cells, the medium was pretreated with or without NAM (15 μM) and Compound C (20 μM) for 30 min. Then, 17β-E2 (0 M or 1 × 10^-7^ M) was added to the chondrocytes for 24 h, followed by treatment with 10 µl of MTT reagent (5 mg/ml), and the cells were incubated at 37°C for 3 h. Then, the cells were incubated with MTT solution for 4 h, and DMSO was used instead of the culture medium. The absorbance was measured at 570 nm wavelength with an RNE-90002 Microplate Reader (REAGEN, NJ, USA). The experiments were independently repeated 3 times.

### Cell Proliferation Assay

The chondrocytes were seeded on the 96-well plates and then pretreated with or without NAM (15 μM) and Compound C (20 μM) for 30 min. They were treated with BrdU and incubated for 2 h, followed by treatment with 17β-E2 (0 M or 1 × 10^-7^ M) for 24 h following the manufacturer’s protocols (Abcam, MA, USA). They were fixed in 4% paraformaldehyde at 4°C for 30 min and then rinsed with PBS 3 times. Finally, a BrdU Cell Proliferation ELISA Kit (Abcam, MA, USA) was used to examine the BrdU incorporation.

### Statistical Analysis

All data were expressed as mean ± standard deviation (SD) from at least three independent experiments. Data analysis was performed using Student’s t test, analysis of variance (ANOVA), and Student-Newman-Keuls *post hoc* analysis where appropriate. Statistical analyses were conducted using SPSS 25.0 software (SPSS, Inc., IL, USA) for Windows. *P* values < 0.05 indicated a statistically significant difference.

## Results

### 17β-E2 Promoted the Expression of SIRT1 in ATDC5 Chondrocytes

**Figure 1:**
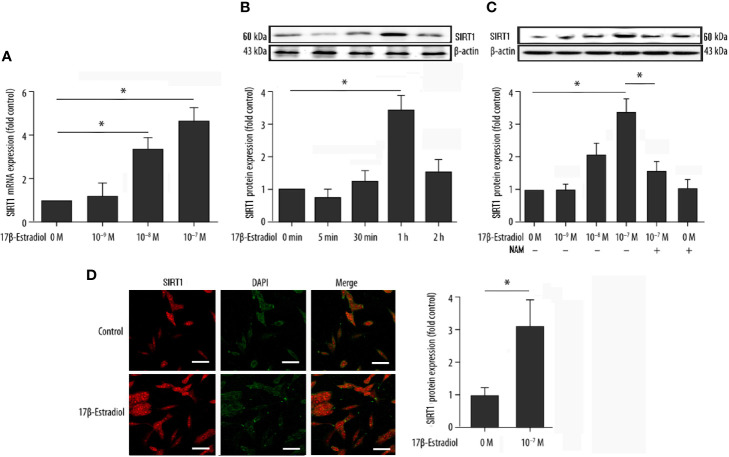
17β-Estradiol (17β-E2) elevated the expression level of SIRT1 in ATDC5 chondrocytes. **(A)** Effect of 17β-E2 on the SIRT1 mRNA expression level in ATDC5 chondrocytes; RT-PCR was performed to demonstrate the expression of SIRT1 mRNA. Results were normalized to GAPDH mRNA. **(B)** Serum-starved ATDC5 chondrocytes were treated with 17β-E2 (1 × 10^-7^ M) for the indicated time (0, 5, 30, 60, and 120 min), and Western blot analysis was applied to show the expression level of SIRT1 protein. **(C)** Serum-starved ATDC5 chondrocytes were pretreated with or without 15 μM NAM for 30 min, followed by treatment with incremental concentrations of 17β-E2 (0 M, 1 × 10^-9^ M, 1 × 10^-8^ M, and 1 × 10^-7^ M). Western blot analysis was performed to demonstrate the expression of SIRT1 protein. β-actin was used as an internal control. **(D)** Confocal immunofluorescence (IF) staining of ATDC5 chondrocytes stained for SIRT1 (red). 4’, 6-diamidino-2-phenylindole (DAPI [blue]) was applied to stain the nucleus. The intensity of SIRT1 was higher in ATDC5 chondrocytes cultured in serum-free medium, which contained 17β-E2. The whole experimental process is described in the *Materials and Methods* section. These experiments were independently repeated 3 times. *P < 0.05 versus the control group indicated a significant difference.

Serum-starved cells were pretreated with or without 15 μM NAM (a selective SIRT1 antagonist) and then treated with various doses of 17β-E2 (0 M, 1 × 10^-9^ M, 1 × 10^-8^ M, and 1 × 10^-7^ M) for 24 h to explore the effect of 17β-E2 on the expression of SIRT1 in ATDC5 chondrocytes. The real-time polymerase chain reaction (RT-PCR) results demonstrated that the expression level of SIRT1 mRNA in ATDC5 chondrocytes increased with a higher concentration of 17β-E2 (**Figure 1A**). At the same time, Western blot analysis yielded similar results (**Figure 1C**). The expression level of SIRT1 protein increased with a higher concentration of 17β-E2, but this effect was blocked by NAM and could not be reversed by 17β-E2 (**Figure 1C**). Similarly, confocal immunofluorescence staining showed that SIRT1 expression was significantly higher in the 17β-E2 group than in the control group (**Figure 1D**). 17β-E2 promoted the expression of SIRT1 mRNA and protein, and 1 × 10^-7^ M 17β-E2 increased the SIRT1 expression to its peak level (**Figures 1A, B** **)**. Hence, 17β-E2 at a concentration of 1 × 10^-7^ M was used in the subsequent experiments. Collectively, the data suggested that 17β-E2 could promote the expression level of SIRT1 in ATDC5 chondrocytes.

### 17β-E2 Promoted the Expression of Mitophagy-Related Proteins in Chondrocytes

**Figure 2:**
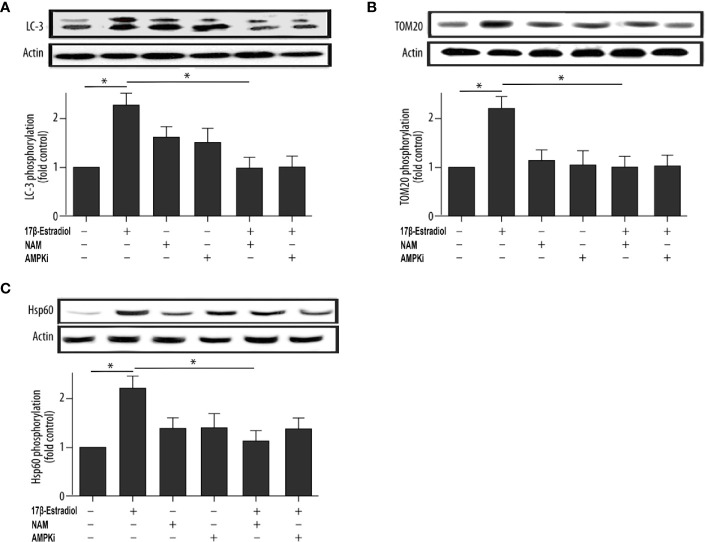
17β-E2 treatment upregulated the phosphorylation level of LC3, TOM20, and Hsp60 in ATDC5 chondrocytes. The chondrocytes were pretreated with 15 μM NAM or 20 μM Compound C for 30 min and then incubated with or without 1 × 10^-7^ M 17β-E2 for 24 h. The phosphorylation levels of **(A)** microtubule-associated protein 1A/1B light chain 3 (LC3), **(B)** translocase of outer membrane 20 (TOM20), **(C)** heat shock protein 60 (Hsp60), and β-actin protein were detected by Western blot analysis. These experiments were independently repeated 3 times. *P < 0.05 versus the control group indicated a significant difference.

This study further investigated the effect of 17β-E2 on the activity of mitophagy-related proteins so as to determine the role of 17β-E2 in mitophagy of ATDC5 chondrocytes and its mechanism. The phosphorylation of LC3, TOM20 (a marker of mitophagy), and Hsp60 (another marker of mitophagy) in ATDC5 chondrocytes was detected using Western blot analysis and compared with that in the control group. The data showed that the phosphorylation of LC3 (**Figure 2A**), TOM20 (**Figure 2B**), and Hsp60 (**Figure 2C**) was significantly higher in the 17β-E2 group than in the control group. Similarly, confocal immunofluorescence staining showed that LC3 expression was significantly higher in the 17β-E2 group than in the control group (**Figure 4**). However, this incremental effect was eliminated in the 17β-E2 + NAM and 17β-E2 + Compound C groups. Collectively, the data suggested that 17β-E2 promoted the expression of mitophagy-related proteins in ATDC5 chondrocytes, but this effect was blocked by NAM or Compound C.

### 17β-E2-Induced Mitophagy Was Mediated by SIRT1

**Figure 3:**
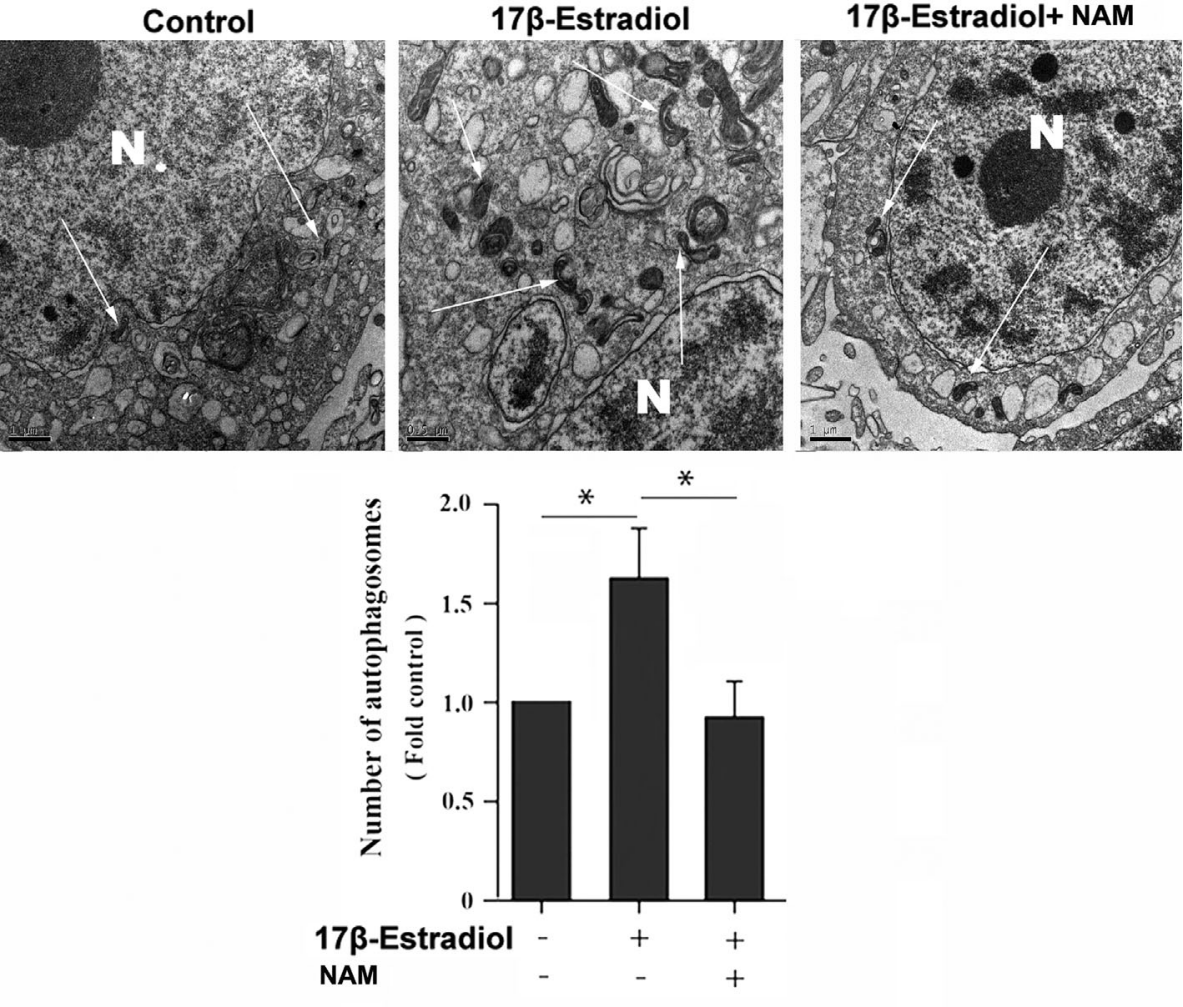
Effect of SIRT1-mediated 17β-E2-induced mitophagy in ATDC5 chondrocytes. Transmission electron microscope (TEM) images indicated more mitophagomes (double-membrane vacuoles) in ATDC5 cells pretreated with 1 × 10^-7^ M 17β-E2 for 24 h compared with those in the control group and 17β-E2 + NAM group. White arrows indicate the mitophagomes (autophagic vacuoles), and the nucleus is represented by the N of ATDC5 chondrocytes. *P < 0.05 versus the control group indicated a significant difference.

**Figure 4:**
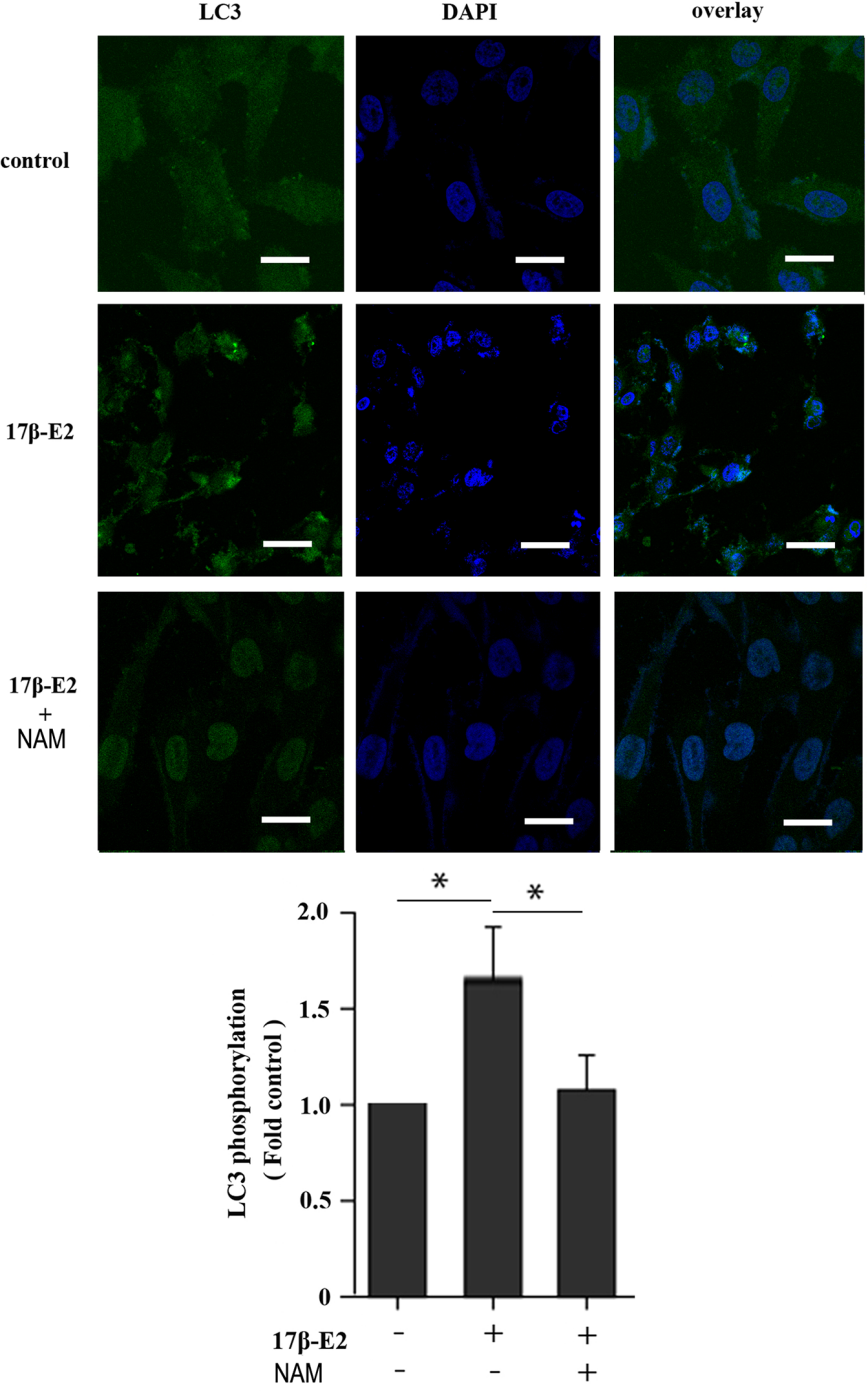
Effect of 17β-E2 and SIRT1 on expression of LC3 in ATDC5 chondrocytes. Confocal microscopy was performed to evaluate autophagy in chondrocytes pre-treated with 17β-E2 or NAM. Nucleus were stained with 4’, 6-diamidino-2-phenylindole (DAPI) (blue). Representative images of confocal immunofluorescence (IF) staining showing the LC3 in chondrocytes. White bar = 50 μm. *P < 0.05 versus the control group indicated a significant difference.

Previous studies reported that mitochondrial autophagosomes (mitophagomes) were double-membrane vesicles, which were considered to be an important structure of mitophagy. Transmission electron microscope (TEM) was regarded as an effective means to detect mitophagy (36). ATDC5 chondrocytes were treated with 1 × 10^-7^ M 17β-E2 for 24 h to determine whether 17β-E2-induced mitophagy in ATDC5 chondrocytes was mediated by SIRT1, and then the characteristics and number of mitophagomes were observed using TEM. The TEM images of mitophagy ultrastructure showed significant differences in mitophagy activity in the three groups (*P* < 0.05). The number of mitophagomes increased significantly in the 17β-E2 group but decreased in the 17β-E2 + NAM group, compared with the control group (**Figure 3**). Confocal immunofluorescence staining showed that the percentage of LC3-positive cells in 17β-E2 group was higher than that in 17β-E2 + NAM group (**Figure 4**). It suggested that 17β-E2 could induce mitophagy upregulation and SIRT1 was an essential component for 17β-E2-induced mitophagy in chondrocytes. In brief, the results demonstrated that 17β-E2 could induce SIRT1-mediated mitophagy upregulation in ATDC5 chondrocytes.

### 17β-E2 Induced Mitophagy *via* the SIRT1-Mediated AMPK/mTOR Pathway

**Figure 5:**
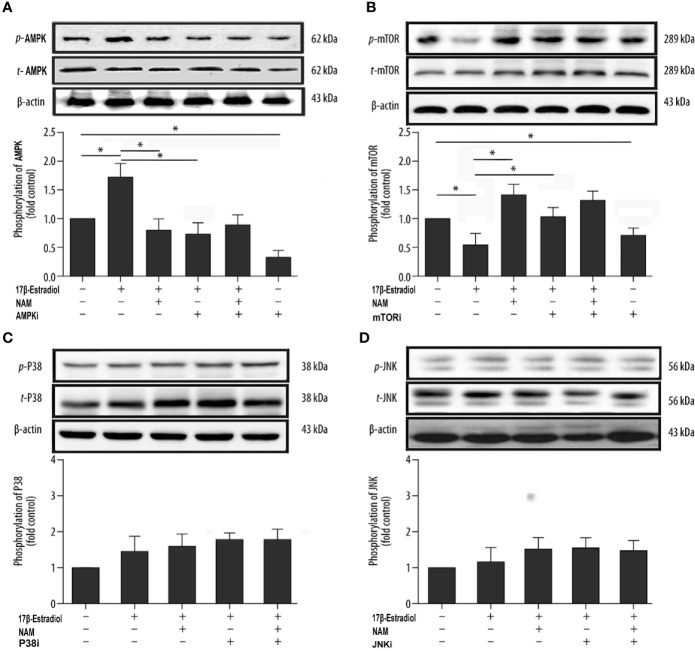
17β-E2 induced mitophagy through the SIRT1-mediated AMPK/mTOR pathway in ATDC5 chondrocytes. The ATDC5 chondrocytes were treated with 17β-E2 (0 M or 1 × 10^-7^ M), with or without pretreatment with NAM, AMPK inhibitor, mTOR inhibitor, p38 inhibitor, and JNK inhibitor. Then, the expression of p-AMPK **(A)**, p-mTOR **(B)**, p-p38 **(C)**, and p-JNK **(D)** in ATDC5 chondrocytes was measured by Western blot analysis. β-actin was used as an internal control to normalize the data. Data are presented as the mean ± SD of three independent experiments. *P < 0.05 versus the control group indicated a significant difference.

SIRT1, AMPK, and mTOR are involved in the modulation of autophagy, which is a significant cellular homeostasis self-regulation mechanism required for longevity, injury repair, survival, differentiation, and pathogen clearance (37). Based on previous results, it was hypothesized that 17β-E2 might induce mitophagy upregulation through the SIRT1-mediated AMPK/mTOR signaling pathway (**Figure 7**). To confirm this hypothesis, chondrocytes were treated with 17β-E2, with or without pretreatment with NAM, AMPKi (Compound C, AMPK inhibitor), and mTORi (S1842, mTOR inhibitor). The phosphorylation of AMPK and mTOR was detected by Western blot analysis. The results showed that p-AMPK levels increased significantly, but p-mTOR expression sharply decreased in the 17β-E2 group compared with the control group (**Figures 5A, B** **)**. Of note, this effect was blocked by the SIRT1 inhibitor and could not be reversed by 17β-E2. Nevertheless, the total AMPK and total mTOR levels had no change in different groups. In addition, NAM (SIRT1 inhibitor) abolished the activation of AMPK by 17β-E2 for 24 h as judged by a lower level of p-AMPK and a higher level of its downstream target p-mTOR. These data suggested that the effect of 17β-E2 on the AMPK/mTOR pathway was mediated by SIRT1 in ATDC5 chondrocytes.

On the contrary, previous studies showed that mitogen-activated protein kinases (MAPKs) were associated with autophagy, including the c-Jun-N-terminal kinase (JNK) and P38 pathways (37). Serum-free chondrocytes were pretreated with or without NAM, JNKi (SP600125, JNK inhibitor), and p38i (SB203580, p38 inhibitor) prior to treatment with 17β-E2 to investigate whether SIRT1 could mediate mitophagy *via* AMPK/JNK and AMPK/p38-MAPK pathways. The Western blot results showed no significant differences in p-p38 and p-JNK levels in different groups, suggesting that the ability of SIRT1 to promote autophagy was not *via* AMPK/JNK and AMPK/p38-MAPK in 17β-E2-treated chondrocytes (**Figures 5C, D** **)**. Taken together, the data revealed that 17β-E2 promoted the expression of SIRT1 in ATDC5 chondrocytes. They also supported the hypothesis that 17β-E2 might induce mitophagy upregulation *via* the SIRT1-mediated AMPK/mTOR signaling pathway.

### 17β-E2 Induced Mitophagy Upregulation to Protect Chondrocytes *via* SIRT1-Mediated AMPK/mTOR Signaling

**Figure 6:**
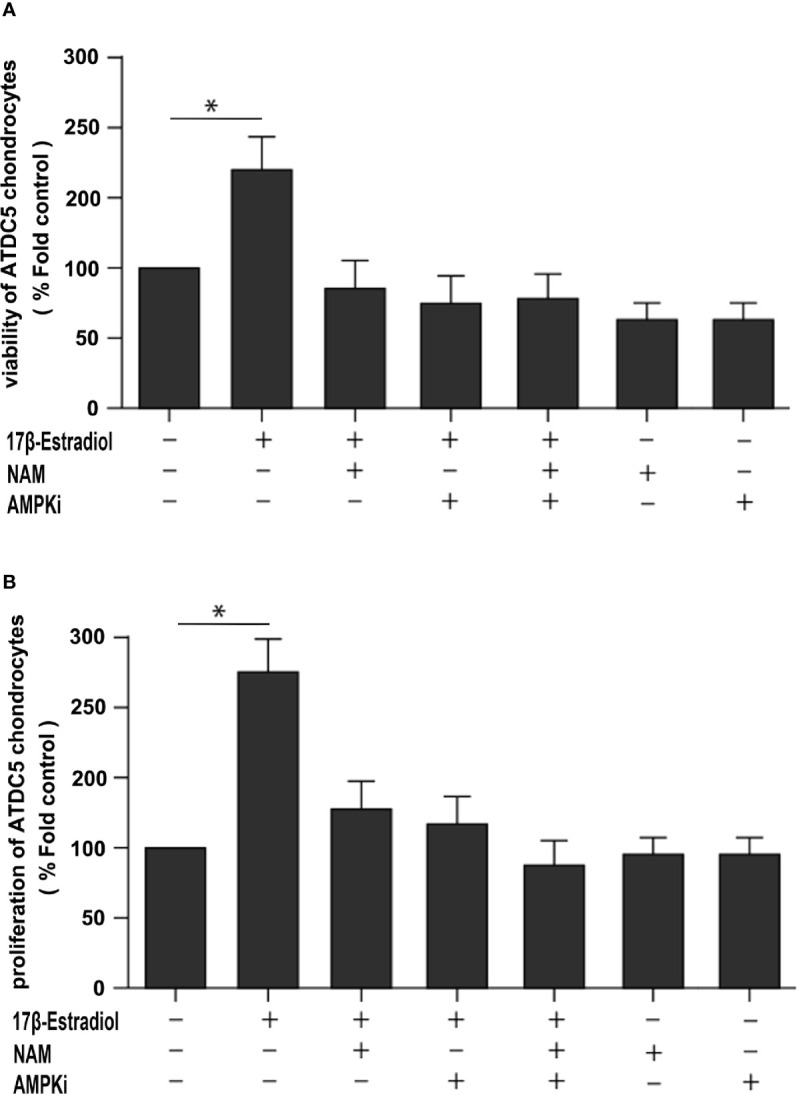
A beneficial effect of 17β-E2 by upregulating mitophagy through the SIRT1-mediated AMPK/mTOR pathway. **(A)** MTT and **(B)** bromodeoxyuridine (BrdU) were used to detect the viability and proliferation of ATDC5 chondrocytes. The cells were cultured in the presence or absence of NAM and AMPK inhibitors for 30 min prior to treatment with 17β-E2 (0 M or 1 × 10^-7^ M) 24 h. These experiments were independently repeated 3 times. *P < 0.05 versus the control group indicated a significant difference.

**Figure 7:**
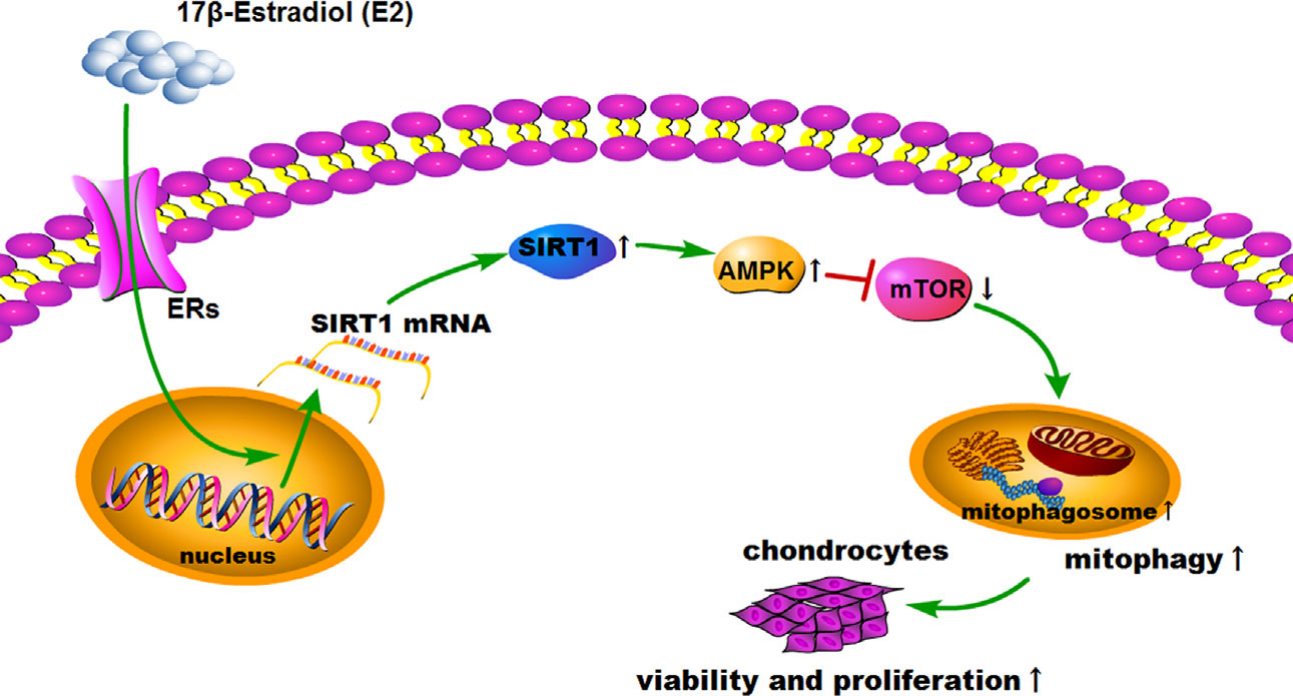
Schematic diagram depicts the proposed and underlying mechanism that 17β-E2 induces mitophagy up-regulation to protect chondrocytes *via* the SIRT1-mediated AMPK/mTOR signaling pathway.

This study further explored whether the protective effect of 17β-E2 in ATDC5 chondrocytes was related to mitophagy upregulation *via* the SIRT1-mediated AMPK/mTOR signaling pathway. The viability of chondrocytes in different groups was measured using the MTT assay (**Figure 6A**). The results showed that the viability of chondrocytes was significantly higher in the 17β-E2-treated group than in the control group, but this effect was abolished in the presence or absence of NAM and AMPKi (Compound C, AMPK inhibitor). Similarly, the bromodeoxyuridine (BrdU) assay revealed that the proliferation increased significantly in the 17β-E2 group, but the effect was blocked by NAM or AMPKi (**Figure 6B**). Together, these data suggested that 17β-E2 could regulate the viability and proliferation through the SIRT1-mediated AMPK/mTOR signaling pathway in ATDC5 chondrocytes. Also, they supported the hypothesis that 17β-E2 might induce mitophagy upregulation *via* the SIRT1-mediated AMPK/mTOR signaling pathway to protect chondrocytes (**Figure 7**).

## Discussion

The present study showed that exposure to 17β-E2 promoted the expression of SIRT1 to positively regulate AMPK and inhibit mTOR, which was involved in the induction of mitophagy upregulation in ATDC5 chondrocytes. Moreover, during this study, we observed that 17β-E2 induced mitophagy upregulation *via* the SIRT1-mediated AMPK/mTOR signaling pathway to promote the proliferation and viability of chondrocytes. Several studies have showed that 17β-E2 and its receptor could promote cell proliferation and viability (38), decrease the cartilage damage in experimental OA models (39, 40). Our findings showed the beneficial effect of 17β-E2 against OA, which might be a potential therapy strategy for OA.

Autophagy is activated under hypoxic conditions and energy stress to provide energy for cells. Also, it is a key regulator of cell-autonomous homeostasis through the clearance of damaged macromolecules and organelles, including mitochondria (41). Mitophagy is the elimination of depolarized and damaged mitochondria. The pharmacological activation of autophagy in chondrocytes has been demonstrated to significantly protect against mitochondrial dysfunction, indicating that mitophagy might function to eliminate damaged and dysfunctional mitochondria in chondrocytes and prevent oxidative stress (41). Research on human chondrocytes suggested that the activation of autophagy was critical in protecting against mitochondrial dysfunction (42).

The relationship between 17β-E2 and mitophagy has become an interesting research field. Increasing evidence indicates that mitochondria are involved in the pathogenesis of OA (43). Mitochondrial dysfunction can contribute to cartilage degeneration in OA. Recently, Xu Kang et al. conducted an experiment on rat OA model chondrocytes and concluded that 17β-E2 could promote cell proliferation in the OA model (44). This finding was in agreement with the results of the present study showing the protective effect of 17β-E2 on ATDC5 chondrocytes. In the present study, 17β-E2 promoted the expression of SIRT1, and 17β-E2-induced mitophagy was mediated by SIRT1. Furthermore, in the presence or absence of NAM, AMPKi treatment significantly blocked the levels of SIRT1 and mitophagy markers. On the contrary, TEM images revealed more mitophagosomes and richer autophagosomes in ATDC5 chondrocytes treated with 17β-E2; but the changes were opposite in the 17β-E2 + NAM group. Correspondingly, the viability and proliferation of chondrocytes increased significantly, as shown by MTT and BrdU results. Taken together, these data suggested that 17β-E2 could induce mitophagy upregulation *via* SIRT1 mediation to protect chondrocytes.

AMPK is an important pathway of modulating cellular energy and metabolic homeostasis. It is vital in the angiogenesis, apoptosis, tumorigenesis, and autophagy. mTOR is one of the main inhibitors of autophagy; it is important in the occurrence and development of autophagy (45). In addition, AMPK, as an upstream protein, decreased the levels of mTOR when phosphorylation increased (25), thus promoting autophagy. Recently, many in-depth studies were conducted on the pathogenesis of OA, revealing related signaling pathways, such as the AMPK/mTOR signaling pathway (46, 47), having a significant impact on OA. The present study showed that 17β-E2 promoted the expression of SIRT1 in ATDC5 chondrocytes, which was consistent with the findings of Mehtab et al. (48). 17β-E2 could mediate the SIRT1 activity, while SIRT1 was involved in the regulation of autophagy in chondrocytes (29, 49). Hence, it was inferred that 17β-E2 might induce mitophagy upregulation through the SIRT1-mediated AMPK/mTOR signaling pathway in ATDC5 chondrocytes. The MTT and BrdU results showed that the cell viability and proliferation in the 17β-E2-treated group increased, but the beneficial effects were blocked in the presence or absence of NAM and AMPKi. These results supported the hypothesis that 17β-E2 induced mitophagy upregulation through the SIRT1-mediated AMPK/mTOR signaling pathway, thus protecting chondrocytes (**Figure 7**). Besides the AMPK/mTOR signaling pathway, we further investigated whether SIRT1 could mediate mitophagy *via* JNK and p38 signaling pathways. Chondrocytes were pretreated with or without NAM, JNKi and p38i for 30 min prior to treatment with 17β-E2 for 24 h (33, 50). These preliminary data suggested that the ability of SIRT1 to promote mitophagy in 17β-E2-treated chondrocytes was not through JNK and p38 pathways.

Despite significant discoveries, this study had certain limitations. First, only one cell line ATDC5 was used for the *in vitro* experiments. Next, the concentration of 17β-E2 *in vitro* was several orders of magnitude higher than that *in vivo*; therefore, it was hard to explain the conditions *in vivo*. Physiologic concentrations of estrogen without serum appeared to have no effect on chondrocytes proliferation or viability. Claassen et al. (51) reported that incubation with physiological dose of estradiol (10^-11^ M) alone did not significantly influence collagen II synthesis in their study of cow chondrocytes. *In vivo*, ovariectomy (OVX) female mice developed significantly severe OA than control females, whereas the severity of OA was back to the level of control females when supplemented with exogenous 17β-E2 (52, 53). In our study, we showed that 17β-E2 induced mitophagy upregulation *via* the SIRT1-mediated AMPK/mTOR signaling pathway to promote the proliferation and viability of chondrocytes *in vitro*. Our results showed the beneficial effect of 17β-E2 against OA, however, further studies are needed.

In this study, we demonstrate that 17β-E2 at a pharmacological concentration (1 × 10^-7^ M) could stimulate the expression of SIRT1 in ATDC5 chondrocytes *in vitro* mainly through the AMPK/mTOR pathway in mitophagy. Moreover, these findings suggested that 17β-E2 induced mitophagy upregulation to protect chondrocytes *via* the SIRT1-mediated AMPK/mTOR pathway. Meanwhile, the data provided a new perspective for 17β-E2 mechanisms in OA and suggested that the SIRT1-mediated AMPK/mTOR signaling pathway was a potential therapeutic target for OA. Even though the roles of 17β-E2 may play the positive effects against aging-related diseases. There are some controversies regarding the effects of 17β-E2 on the regulation of SIRT1 in the vasculature. Lee et al. (54) reported that the decline in SIRT1 levels and function of the vascular smooth muscle cells (VSMCs) treated with estrogen suggested a possible detrimental role for estrogen in vascular health. In vivo and *in vitro*, 17β-E2 was able to protect cardiomyocytes from AngII-induced injury with a profound upregulation of SIRT1 and activation of AMPK (29). Our observations may partially explain the positive impact of 17β-E2 replacement therapy on bone metabolism disease. Improved understanding of the molecular actions of 17β-E2 is required, and more studies are needed.

## Data Availability Statement

The original contributions presented in the study are included in the article/**Supplementary Material**. Further inquiries can be directed to the corresponding authors.

## Author Contributions

RM, LG, and XY contributed to conceive and design the project. RM, PL, GY, and TJ contributed to prepare libraries, perform experiments and analyses the data. RM and GY wrote the manuscript. RM, LG, and XY contributed to make manuscript revisions and funds collection. All authors contributed to the article and approved the submitted version.

## Funding

This work was supported by the National Natural Science Foundation of China (Grant No. 81971322, 81473707) and the Special Innovation Fund for Postgraduates of Jiangxi Province (No. YC2020-B044).

## Conflict of Interest

The authors declare that the research was conducted in the absence of any commercial or financial relationships that could be construed as a potential conflict of interest.
