# Supplementary material for: 17β-Estradiol Induces Mitophagy Upregulation to Protect Chondrocytes via the SIRT1-Mediated AMPK/mTOR Signaling Pathway
Source: Front Endocrinol (Lausanne). 2021 Feb 3;11:615250. doi: 10.3389/fendo.2020.615250 (PMC7888342; doi:10.3389/fendo.2020.615250)
Supplement: Supplementary file 1 [file DataSheet_1.pdf]

frontiers

Supplementary Material

Supplementary Figures

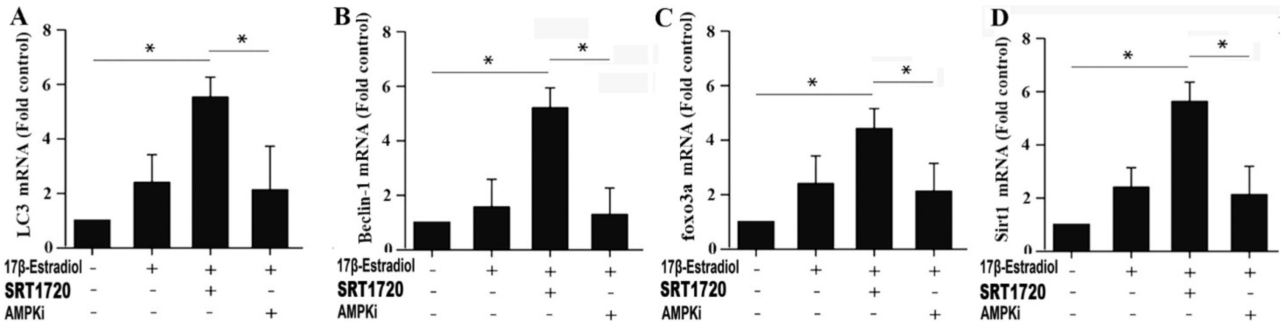

Supplementary Figure 1 17β-E2 treatment promoted the mRNA expression level of LC3 (**A**), Beclin-1 (**B**), foxo3a (**C**) and SIRT1 (**D**) in ATDC5 chondrocytes. The cells were pretreated with SRT1720 (SIRT1 activator) or 20 μM Compound C (AMPK inhibitor), and then incubated with or without 1×10^{-7} M 17β-E2 for 24 h. The mRNA expression level of microtubule-associated protein 1A/1B light chain 3 (LC3), Beclin-1, forkhead box O 3a (foxo3a) and SIRT1 were detected by RT-PCR. GAPDH was used as an internal control to normalize the data. These experiments were independently repeated three times. Data are presented as the mean ± SD. *$P < 0.05$ versus the control group indicated a significant difference.
